# Low-Cost Air Quality Monitoring Tools: From Research to Practice (A Workshop Summary)

**DOI:** 10.3390/s17112478

**Published:** 2017-10-28

**Authors:** Andrea L. Clements, William G. Griswold, Abhijit RS, Jill E. Johnston, Megan M. Herting, Jacob Thorson, Ashley Collier-Oxandale, Michael Hannigan

**Affiliations:** 1 Office of Research and Development, Environmental Protection Agency, Research Triangle Park, NC 27711, USA; 2 Department of Computer Science and Engineering, University of California San Diego, La Jolla, CA 92093, USA; wgg@cs.ucsd.edu; 3 Office of Chief Scientist, Environmental Defense Fund, San Francisco, CA 94105, USA; arudrapatna@edf.org; 4 Department of Preventive Medicine, University of Southern California, Los Angeles, CA 90089, USA; jillj@usc.edu (J.E.J.); herting@usc.edu (M.M.H.); 5 Department of Mechanical Engineering, University of Colorado Boulder, Boulder, CO 80309, USA; jacob.thorson@colorado.edu (J.T.); ashley.collier@colorado.edu (A.C.-O.); michael.hannigan@colorado.edu (M.H.)

**Keywords:** air quality, low-cost sensors, data standards, data management, community-based organizations

## Abstract

In May 2017, a two-day workshop was held in Los Angeles (California, U.S.A.) to gather practitioners who work with low-cost sensors used to make air quality measurements. The community of practice included individuals from academia, industry, non-profit groups, community-based organizations, and regulatory agencies. The group gathered to share knowledge developed from a variety of pilot projects in hopes of advancing the collective knowledge about how best to use low-cost air quality sensors. Panel discussion topics included: (1) best practices for deployment and calibration of low-cost sensor systems, (2) data standardization efforts and database design, (3) advances in sensor calibration, data management, and data analysis and visualization, and (4) lessons learned from research/community partnerships to encourage purposeful use of sensors and create change/action. Panel discussions summarized knowledge advances and project successes while also highlighting the questions, unresolved issues, and technological limitations that still remain within the low-cost air quality sensor arena.

## 1. Introduction

In the United States, air quality has traditionally been measured according to a metric established by the United States Environmental Protection Agency (USEPA) using equipment that implement a federal reference method (FRM) or federal equivalent method (FEM). These devices cost tens of thousands of dollars and require significant infrastructure and trained personnel to operate [1]. Within the last ten years, miniaturization and other technological advances have brought to market a number of low-cost (<$2500) sensors designed to measure atmospheric particles and gases. Although sensors cannot replace traditional FRM/FEM monitors, these sensors have created new opportunities for broadening access to ambient air quality monitoring for applications such as personal health and sub-regional air quality assessment [2,3]. Residents in Environmental Justice communities are particularly interested in using sensor technology to gather neighborhood-level data to illustrate the impact of specific emissions sources and magnitude of air quality issues affecting their communities.

Efforts relating to the application of this new generation of low-cost sensors have taken several forms. While much has been invested in the development of low-cost ambient air quality sensors, research to identify appropriate and purposeful use of sensors and networks and develop data analysis and visualization tools to process and interpret collected data is ongoing. In order to increase sensing accuracy, studies have sought to characterize how low-cost sensors respond under changing environmental conditions.

Accuracy and reliability have become unifying concerns. Sensor system development and implementation have been hampered by the complex nature of low-cost gas and particle sensor responses. Some sensors have exhibited vexing manufacturing variations and sensor sensitivity to environmental factors like temperature, humidity, and barometric pressure has proven difficult to model when the conditions range widely. The response of low-cost sensors has been shown to change as they age, in connection with how long they have been in operation. Issues with sensor drift have necessitated frequent recalibration, reducing their cost advantage. In addition, low-cost sensors have also proven sensitive to enclosure air exchange rates, complicating mobile deployments, which are a popular application of low-cost sensors because of their small size and low power requirements [4]. Sensors can be slow to respond to changes in pollutant levels, causing pollutant spikes encountered in mobile deployments to be underestimated. Finally, air quality sensors are often marketed for one pollutant, but exhibit cross-sensitivity to other pollutants. Ongoing research is investigating a number of sensors to identify cross-sensitivities and working towards resolution and quantification of individual gas species using advanced calibration techniques and comparison to FRM/FEM measurements [5,6,7,8,9,10,11,12]. Keeping abreast of this technology sector is difficult at best given that new sensors or versions are coming to market seemingly every day and our ability to evaluate the technology requires painstaking research.

All of these efforts have resulted in valuable lessons and the MetaSense research team [13] convened this workshop with the leading practitioners in the field of low-cost air sensing to share insights and discuss open problems. The community of practice included academic researchers, industry professionals, non-profit groups, community-based organization, and regulatory agencies. The workshop was organized into four panels guided by the following topics: (1) best practices for deployment and calibration of low-cost sensor systems, (2) data standardization efforts and database design, (3) advances in sensor calibration, data management, and data analysis and visualization, and (4) lessons learned from research/community partnerships to encourage purposeful use of sensors and create change/action. The following summarizes the workshop’s discussions among panel experts and the community of practice and shares insights from two community-based organizations gathering air quality data in an effort to improve their communities.

## 2. Panel 1: ‘Best Practices’ for Deployment and Calibration of Low-Cost Sensor Systems

Significant effort has been devoted to exploring the use of low-cost air quality sensors. The low-cost and small size of these devices make them very attractive for increasing the spatial coverage of existing networks, deploying sensors in urban locations where small footprints are desirable, and mobile or semi-mobile sampling schemes. During Panel 1, panelists Andrea Polidori (South Coast Air Quality Management District, Diamond Bar, CA, USA), Ron Cohen (University of California, Berkeley, CA, USA), Jonathan Thornburg (Research Triangle Institute, Durham, NC, USA), and Angelo Bianchi (AQMesh, Stratford-upon-Avon, UK) discussed the exploratory research done to understand the appropriate use of sensors, evaluation efforts being conducted by a variety of institutions, current sensor performance expectations, the state of calibration research, and recent deployment efforts. The overall conclusion was that this field is in transition and more work and funding is needed to continue to realize the full potential of low-cost air quality sensors.

### 2.1. Categorization and Use of Sensor Systems

There appears to be a common misconception that low-cost air quality sensors are capable of measurements comparable to FRM/FEM measurements. Experience proves this is not the case with some sensors showing no correlation to FRM/FEM measurements, while others show reasonable correlations (r^2^~0.7) [14,15]. The USEPA is not currently entertaining sensor applications for FEM consideration. In addition to poor correlations, sensors often have greater uncertainty. Thus, many sensors fall into an undefined space somewhere between qualitative educational and regulatory compliance measurements and future work may further define this space.

Although low-cost air quality sensors, in their current state, cannot be used for regulatory or compliance purposes, there are a number of appropriate and useful application for these low-cost tools [16,17]. Sensors may not be able to report a sufficiently precise or accurate pollutant concentration to replicate FRM/FEM measurements but some correlate fair to reasonably well (r^2^ = 0.4–0.8) [14,15]. Those that correlate can be used to supplement existing monitoring networks to increase spatial coverage and fill knowledge gaps. They can be used to measure smaller scale variations in spatial concentration or determine how a suspected source may be affecting a nearby community, frequent concerns of community-based organizations. Education and developing air quality awareness are natural applications of sensor technologies and provide a means by which citizens and students might learn about air quality issues, sources impacting air pollution, and variations in air quality in various environments such as work, home, and outdoors. Personal exposure monitoring is another emerging and exciting application for sensors, especially for individuals who are more sensitive to air pollution. Personal exposure monitoring may help an individual make decisions about the timing and location of daily activities like commuting and exercise, based on air quality data. Whether or not a particular sensor is being used appropriately is application dependent—the sensor model, the calibration/deployment procedures, and even data processing and interpretation should all be motivated by the research question.

### 2.2. Sensor Evaluation

Choosing an appropriate sensor is an important first step in any data collection effort. To date, three notable programs have been established to characterize the performance of low-cost air quality sensors and to make the results of such evaluations available to all potential users. In each case, evaluations are done objectively and the evaluating programs often outright purchase the sensors to ensure integrity. Sensors are often evaluated under real-world outdoor field conditions where sensors are placed alongside the traditional FRM/FEM equipment to which their data are compared. Laboratory testing is also employed for evaluations and involves exposing sensors to known pollutant concentrations within an environmental chamber. Evaluations may include variable temperature and relative humidity conditions as well as introduction of known or possible interfering pollutants. These results should be consulted in the search for an appropriate sensor.

The Air Quality Sensor Performance Evaluation Center (AQ-SPEC), operated by the South Coast Air Quality Management District (SCAQMD), was established in 2014 to evaluate the accuracy and usability of commercially available low-cost air quality sensors. At present, evaluations focus primarily on turn-key products that are ready for immediate deployment and/or operation. All sensors evaluated in this program are operated outside in southern California field conditions, and reasonably performing sensors are also tested under controlled laboratory conditions with varied temperature and relative humidity conditions. Sensor evaluation reports and details about testing protocols are available on the AQ-SPEC website located at www.aqmd.gov/aq-spec [15].

The USEPA Office of Research and Development (ORD) also conducts evaluations of low-cost air quality sensors and the evaluations results are one of the many resources made available through the USEPA Air Sensors Toolbox located at www.epa.gov/air-sensor-toolbox [14]. The USEPA has undertaken a number of sensor evaluation efforts under outdoor field and controlled laboratory conditions. More field evaluations are undertaken than lab evaluations and most have been conducted at the Ambient Air Innovation Research Site (AIRS) test platform at the Research Triangle Park location in North Carolina. In addition to turn-key products, USEPA has also evaluated some component based sensors and have incorporated such sensors into a number of devices including the Village Green Stations, AirMappers, and several versions of the Citizen Science Air Monitors (CSAMs). These devices, and a small number of turn-key products, have been operated and evaluated over the course of several small to mid-sized field deployments connected to USEPA projects all over the country [14].

The Joint Research Center (JRC), as the European Commission’s Science and Knowledge Service, has also conducted research evaluating low-cost air quality sensors via testing under controlled laboratory conditions in a state-of-the-art chamber and outdoor field deployments. Laboratory evaluations have focused on component based sensors, calibrations, and long-term experiments to give insight into long-term performance and drift. Field deployments have investigated normalization techniques too. Information about the chamber, testing protocols, sensor evaluations, and field deployments are mainly found through reports and in the scientific literature [11,18,19]. Additionally, a number academic researchers have published papers sharing lab evaluations [20] or field performance data on a limited selection of sensors [21,22,23,24,25].

Sensor performance evaluations have been extremely helpful for practitioners. However, under the current paradigm, the evaluating institutions pay for the sensors they evaluate, which is likely to be unsustainable in the long run with a rapidly changing marketplace and increasing costs as sensor systems get more complex. Debates about the path forward are ongoing and options include third party evaluation, sensor certification, or a program which would encourage manufactures to self-evaluate. Although these discussions are happening now, it is important to note that such programs come with significant investments of time (e.g., developing test methods for each pollutant, getting manufactures on board) and money (e.g., start-up, program maintenance) and are likely to take many years to develop.

### 2.3. Current State of Sensor Performance

The sensor evaluation efforts described have provided insight into the current state of sensor performance and have elucidated areas where further research and development is needed. Evaluations show that currently available particulate matter (PM) sensors exhibit reasonable performance (select sensors approaching 0.7 < r^2^ < 0.9) [15]. Evaluations found that most PM sensors have minimal downtime, moderate inter-sensor variability, and show reasonable correlation with FRM/FEM measurements, although calibration and normalization is still needed. Many show temperature and humidity effects, especially at high humidity, and under-report at very high (>200 μg/m^3^) concentrations [26,27]. Additionally, most sensors cannot detect very small particles (lower cutoffs between 0.3 and 1 μm) and will miss ultrafine particles and smoke [28]. Some work is ongoing to develop sensors capable of measuring particles in this small size range. Most of the current sensors detect particle counts rather than particle mass and must use an algorithm to report PM mass concentrations [28].

Evaluations show that gas-phase sensors exhibit acceptable data recovery but have more inter-sensor variability than PM sensors. When carbon monoxide (CO), nitrogen dioxide (NO_2_), and ozone (O_3_) are measured alone in a laboratory setting under controlled conditions without confounding gas species present, sensors exhibit reasonable to good correlation with FRM/FEM measurements (0.8 < r^2^ < 0.99) [15,29,30]. Sensors that are cross-sensitive to multiple pollutants show low correlations with FRM/FEM measurements when operated in the field environment where a mixture of pollutants is present (0.3 < r^2^ < 0.9) [15,30]. Temperature and relative humidity (RH) have a larger effect on gas phase sensors leading to decreased sensitivity in high RH conditions and degradation over time [6,31]. Therefore, repeated field calibration of gas phase sensors is needed and is further discussed in the next section. To date, ambient concentrations of sulfur dioxide (SO_2_), hydrogen sulfide (H_2_S), methane (CH_4_), and volatile organic compounds (VOCs) prove extremely difficult to quantify despite sensors that advertise sensitivity to these species.

### 2.4. Sensor Calibration

Sensor performance evaluations have indicated a need to calibrate sensor response if one wishes to compare one sensor’s data to that of another sensor or to nearby regulatory monitoring data. Field normalization of sensor signals that have been collocated with FRM/FEM measurements is the most common method of calibrating sensor measurements. Linear regression is commonly used to normalize sensor signals to reference measurements, but there is no evidence that these correlations are transferrable to different locations. Environmental factors such as temperature, relative humidity, relative concentration of confounding pollutants, and particle sources and variation in particle size are all known to affect sensor response, so it is not surprising that these variables also change how sensor measurements compare with the reference.

Researchers are exploring different methods of calibrating sensors against reference measurements motivated by the known presence of complex nonlinear and cross-sensitive behavior of sensors. Field normalization techniques that attempt to address these complex behaviors include multi-linear regressions, non-linear multi-variate models, and machine learning [8,11,12,31,32]. These methods may make calibrations more transferrable between regions because they consider many of the factors known to influence sensor performance, though model extrapolation is a concern. Therefore, it will be important to calibrate over a wide range of environmental variables and pollutant concentrations. Future experiments may investigate if such calibrations can be performed in a laboratory setting [8]. Methods for dealing with sensor aging, which can cause a range of issues from drift to sensor failure, are still largely underdeveloped.

Beyond field normalization to reference measurements, researchers have used their understanding of atmospheric chemistry to add another level of validation to the data produced by sensors. There is great potential for researchers to develop rules based on atmospheric chemistry/physics to ‘check’ sensor data and to share these resources with users. For instance, Ron Cohen shared that his group has been using VOC + NO_2_ ozone formation chemistry to check some of their sensor data. Briefly, O_3_ concentrations should fall to zero at night, if there is any NO present so nighttime sensor readings may point to a bias within the sensor measurements and monitoring changes in this minimum concentration may help detect sensor drift or more dramatic shifts in sensor performance.

### 2.5. Sensor Network Deployment

Numerous sensor deployments have been conducted in recent years ranging from residents investigating air quality in their homes or neighborhoods, to small networks looking at community-level concentrations, to large sensor networks covering cities or regions [10,23,33,34,35,36,37,38]. Increasing network size leads to increasing complexity and exponentially increasing costs and effort for data analysis and visualization.

When deploying sensors for data collection purposes, there are a number of factors to consider. The EPA’s Air Sensor Guidebook [17] may be a helpful resource to those designing a data collection effort using sensors. The following represent key considerations and ‘best practice’ recommendations.

- The research question needs to drive proper sensor selection. Consult evaluation reports during the sensor selection process to better understand how a sensor might be expected to perform given the environment and expected pollutant concentrations.
- Calibration is key to any sucessful deployment. Although researchers are still investiaging a number of ways to calibrate sensors (Section 3.4), collocation of all sensors with nearby FRM/FEM is still an essential best practice. At a minimum, this should be done before any sensor deployment or field study. Repeating the procedure after a deployment will help quantify sensor drift and help bound uncertainty. Long-term deployments often rotate sensors through several short collocation periods to continuely monitor for drift and sensor failure.
- Sensor failure and replacement is a concern especially for successful long-term deloyment. Evaluation efforts have noted significant variation and failure of new sensors in the low-cost price range. Early deployments noted pre-mature failures and indications of short sensor lifetimes with declining performance within the 1st year of use. Users should purchase additional sensors to complete the deployment plan and continually monitor sensors for failure and declining performance.
- The research question and pollutant of interest should govern the size and siting of the sensor network. For instance, if the question involves how air pollutant concentrations vary in the outdoor and indoor environment, a small number of sensors may be needed and siting criteria would include considerations like weather, ventilation, sources, and obstructions. If however, one would like to reliably monitor concentrations over a large area, sensor siting is still important but so is sensor redundancy, pollutant variation, and sensor density within the network.

Data collection and management is a not a trivial matter, especially as the size of a sensor network deployment grows and the data is collected more frequently. Panels 2 and 3 of the workshop were convened to discuss data issues and those discussions are detailed in the following Section 3 and Section 4.

## 3. Panel 2: Data Standardization Efforts and Database Design

The low-cost sensor revolution has been making air quality sensors affordable and available to large populations and community-based organizations. Users come from a variety of backgrounds and have varied objectives. The number of deployed sensors appears to be increasing over time. Currently, data from low-cost air quality sensors comes in a variety of formats sometimes without data labels, units, or metadata to easily understand and process the available information or to compare one dataset to another. Panelists Abhijit RS (Environmental Defense Fund, San Francisco, CA, USA), Andrea Clements (U.S. Environmental Protection Agency, Research Triangle Park, NC, USA) and Michael Hannigan (University of Colorado, Boulder, CO, USA) discussed the need for a harmonized approach to data management. The group discussed the value in developing and adopting data standards.

### 3.1. Data Standardization

A variety of low-cost air quality sensors and sensor systems are presently available. These sensor systems may measure one or more pollutants and/or environmental parameters, employing one of a vareity of measurement techniques. Some sensor systems include onboard algorithms to transform raw data signals into pollutant concentrations. Each uses its own data structure to capture, store, and publish the data.

In order to efficiently store and process large volumes of data sourced from disparate sources, all the incoming data should be representable in a uniform and common structure and format. In practice today, this requires data transformation in order to integrate data from various sources. The idea of data standards plays a very important role in developing a large-scale data management system. Receiving data from sensors in standard data formats would save a lot of time and effort for everyone involved. If sensor system developers adopt data standards, in terms of both data formats and data quality (for example by reporting confidence intervals along with pollutant concentrations), deploying new sensors in the field could become easier by reducing the technical burden on the user and expanding the utility of the measurements they record.

Data format standardization in the air quality domain includes date and timestamp formats, standardized definitions of terms including pollutant names, units of measurement for pollutant concentrations and their interfering factors like meteorological parameters, and a minimum set of data elements to be recorded by the sensors and stored by the backend data system. It also includes data transfer protocols and file formats used for data exchange.

Sensor data currently exists in various formats—comma delimited (CSV) files, XML and JSON formats, database tables, PDF files, etc. Some of these files have headers indicating what is contained in each field or column and some don’t. The date and timestamp in these files may or may not have a timezone designator; they may not take daylight saving changes (DST) into account; some may represent timestamps in UTC while others will report in local time. Sometimes, a date and timestamp is not reported at all. Additionally, the units of measurement vary among these datasets; some sensors report particulate matter (PM) concentration in mass (e.g., μg/m^3^) and others in particle count. The data elements (fields or columns) contained in these datasets vary widely; some files have raw sensor signal or pollutant concentration measurements only while others include statistical summaries like mean and median alongside sensor measurements. Other issues connected to data quality include field duplication, data duplication, unexpected insertion of text character strings, data gaps, and irregular data reporting. Uniform procedures for addressing all of these challenges in every dataset would make it easier to integrate the data in order to perform analysis across data sets.

### 3.2. Air Quality Data Platform

Low-cost sensors can provide data with very high spatial and temporal resolution, which is not easily achieved with conventional instruments. Researchers and academics have been collecting air quality data for decades; in recent years, community organizations have been deploying sensor networks in their neighborhoods to monitor their local air quality and citizen scientists have been using sensors to learn about air quality in their immediate surroundings. However, most of this data is only available to the people who collected the data, and generally not available to a larger audience. These siloed data stores limit the extent to which data analytics can be performed on air quality data. Combining all these datasets and providing a framework amenable to sophisticated analysis would faciliate better understanding of air quality in many places and on many scales. This gained information could help influence behavioral changes that result in improved environmental protection and human health.

There is a need to develop a schema that faciliates air quality data aggregation and sharing. Such a schema could consist of a scalable cloud-based infrastructure, which could provide the capabilities for users to run their computations and analyses instead of downloading data to their local systems for processing. A centralized system could catalyze development of software tools to analyze and visualize the data and make them available to all the users. Air quality researchers and sensor developers could look at wide varieties of pollutant concentration data in concert with factors like meteorology, land use, traffic, and emission sources that affect air quality. Community organizations and citizen scientists would be able to compare various neighborhoods and develop science driven policy recommendations founded on data. The data platform would be in a position to connect with other systems that host data relevant for air quality analyses like health informatics, real estate market, urban planning, emission inventories, and water quality; thereby, expanding the scope of use.

### 3.3. Bridging the Data Gap

The Environmental Defense Fund has convened the Air Sensor Workgroup (ASW), a broad-based group with participants from state and federal government, academic institutions, sensor developers, and other organizations and stakeholders interested in making air quality data open and Findable, Accessible, Interoperable, and Reusable (FAIR). The main objective of the ASW is to enable easy and efficient access to large volumes of air quality data for the common good. To achieve their vision, they developed Date and Timestamp Guidelines and are working on other relevant data standards. They are also developing a data platform to host and publish data collected from low- and medium-cost air quality sensors globally. The ASW does not have any commercial interests and the software and tools developed by them will be released as open source software and will be publicly available at no cost to the users. The ASW encourages users to leverage this data platform to make advances in auto-calibration of sensors and support scaling the sensor deployments in addition to other potential uses. More information about the ASW is available at www.edf.org/asw [39].

### 3.4. Future Needs and Directions

The air quality community needs to move away from qualifying the data as good or bad, and toward characterizing the exact qualities of the sensor data, including confidence in pollutant concentrations. Air quality measurement data should be supplemented with metadata. The data platform will need to be flexible with limited optionality to keep it simple for users. Some basic data quality validations could be performed by the data platform, but it will be up to the end users to determine whether the quality of data is good enough for their particular use.

The sensor calibration details are currently not published widely. This makes researchers wary of the reported measurements. That leads to additional testing and potential recalibration by advanced users. Additionally, air sensors may behave differently under lab conditions and field conditions, which may need to be taken into consideration while calibrating. Hence, providing more information about the out-of-the-box calibration will not only expedite the use of sensors but also create opportunities for improving the calibration methods and scalability of deployment.

One of the important questions is how an open-access data platform might impact local communities and environmental justice issues. Such a data platform may be used to develop products and services, and monetize them. The data by itself may not be monetized but the tools to process and visualize the data could be; the results of data analytics and the corresponding findings could find monetary value as well. While this may not financially benefit the data owners who contributed data to the data platform, a concern of some groups, it certainly helps to advance science and there are potential indirect benefits that data contributors might reap over time. An open-access data platform would allow researchers to perform analyses and then share the results with other platform users. Community groups could use those case studies to guide local action. Community groups may also be able to post their data and solicit assistance with analyses to develop actionable insights. Health scientists and other may be able to combine personal air quality exposures to health outcomes. Eventually, air quality data could be as widely available and interpretable as traffic or meteorological data. An open-access data platform might also lead to a variety of analyses and interpretations; some of them could seem contradictory. This may open up channels for further communication among the researchers and analysts, and might help in advancing science. Negative impacts have yet to be defined.

### 3.5. Summary

Sensor and sensor system developers and users conforming to data standards could facilitate the aggregation of data, making it possible to create a larger, richer dataset which could lead to the discovery of new insights. A common data platform could open up opportunities to integrate data from global sources leading to development of data products and applications that can help users understand air quality at the neighborhood scale. Given this vision, establishing data standards and complying with them is critical to harnessing value from the data measured by low-cost sensors.

## 4. Panel 3: Advances in Sensor Calibration, Data Management, and Data Analysis and Visualization

During Panel 3, panelists Michael Heimbinder (HabitatMap, Brooklyn, NY, USA), Sanjoy Dasgupta (University of California, San Diego, CA, USA), Nicholas Masson (Qsense Inc., Boulder, CO, USA), and Mark Potosnak (DePaul University, Chicago, IL, USA) posed several questions to guide the discussion regarding sensor calibration and data management, analysis, and visualization. The subsequent discussion focused on five key issues outlined and summarized in this Section. Despite outstanding data calibration, quality, and validation issues, participants agreed that there is great value in the data collected by sensors but that this data must be used wisely and with caution. Users were encouraged to collect supplemental data (e.g., metrological data, co-pollutants concentrations, traffic and other observational data) that might help in subsequent data interpretation efforts. Researchers were also encouraged to be open and honest in setting expectations and in explaining the appropriate use and current limitations of sensor technology. Repeatedly, community organizers mentioned the need for effective infographics and data visualization tools to help share data, interpret the results, and educate the public.

### 4.1. Data Quality

As discussed in Section 2, data from low-cost sensors are not equivalent to data from FRMs/FEMs, but rather than thinking of sensor data as “good data” if it compares well to FRMs/FEMs, it might be better to consider if data is “good enough” for the intended objective [17]. For instance, to monitor spatial variation, the paramount consideration is that sensor measurements are comparable to one another. Sometimes, another factor (like how the body responds to a pollutant concentration) may have more uncertainty than the concentration measurements allowing for more flexibility in the sensor uncertainty. Thus, the necessary quality of the data should be considered in the study design process.

Quantification of uncertainty or confidence interval is essential for understanding and using sensor data. Generally, uncertainty is defined through collocation with FRM/FEM instruments, but statistical modeling may help determine the appropriate confidence intervals. The interval should fully capture the uncertainty in the data and the width of this interval can help determine the usefulness of the data. Researchers should be sure to consider whether the measurement uncertainty is driven by the sampling environment, systemic biases, or random error.

Network deployments may alter the data quality questions. Looking at the sensor data in aggregate may render smaller errors unimportant. Environmental factors can significantly influence sensor performance and are likely to remain important. Information about traffic and expected sources may also be helpful in interpreting data. When considering data in aggregate, researchers can look for similar behavioral patterns among a number of sensors to verify changes and may be able to identify or confirm pollution sources. Although this approach may be helpful, care should also be taken not to exclude interesting data. Data that may seem to be outliers may actually be a signal deserving of future investigation.

### 4.2. Supplemental Data Collection

As previously mentioned, environmental factors such as temperature, relative humidity (RH), and the concentration of co-responsive pollutants are all known to affect sensor response. At a minimum, it is important that any data collection effort measure these essential variables. This realization has led to a rise in the development multi-sensor instruments (boxes, pods, systems, etc.). Many of the commercial instruments on the market today are attempting to measure all of these parameters and leverage the instruments to make as many measurements as possible. In many cases, the increased complexity of these instruments takes them from the low-cost sensor realm into a more expensive price range ($2000–$15,000), which also makes them more difficult for communities and citizen scientists to afford, especially if a large distributed sensor network deployment is needed to address research questions.

It is worth noting that temperature and RH measurements also have caveats. Many metal oxide and electrochemical sensors respond to temperature and RH, so measurements of these environmental variables in the air mass directly adjacent to the sensors (within the sensor enclosure if one is used) is very important. Some enclosures are not designed to dissipate heat and temperatures in their interior can differ greatly from the outdoor environment. However, the ambient temperature and RH also influence the chemistry that can affect levels of atmospheric pollutants. Thus, both measurements are important and care should be taken in designing sensor enclosures to minimize the difference between enclosure and ambient measurements.

As the community begins to consider aggregating sensor measurements to make them useful beyond the initial intended use, additional supplemental data may also prove important in interpreting the results. It is difficult to know what might be important when starting a small-scale study and the needs will vary depending on both the level and scope of analysis undertaken. For instance, information about the sensor (make, model, serial number, purchase data, time in service, etc.), position (GPS coordinates), results of collocation efforts, and the calibration equation used are essential. Environmental factors such as temperature, RH, pressure, dew point, wind speed, wind direction, and solar radiation may all assist in understanding sensor response and interpreting variations within the data. Urban or near-roadway data interpretation may benefit from noise/sound data, traffic count, traffic pattern, and vehicle fleet information. Source inventories and source locations may be especially important in interpreting data near sources with episodic and transient behavior. Satellite data may help elucidate the influence of regional sources like wildfire or dust. Unfortunately, it is often impossible to collect all of this information during the course of a data collection effort, often due to cost, but some of this information may be available from other nearby sources (e.g., a weather station, local government website) or a previous study may have many of the same measurements that could give ballpark estimates. Often, researchers involved in a data collection effort will know the best sources for supplementary information and listing them in metadata for future reference would be helpful.

### 4.3. Working with Communities

It is important to understand that many communities are seeking assistance in further understanding their lived experience. They often look for scientific research partners to guide them in collecting and interpreting data. More discussion about these types of partnership and needs are discussed in Section 5.

Communities of all types and scales (neighborhoods to cities to states) are interested in collecting air quality data using sensors but may not be prepared to handle data calibration issues or the vast amount of data that comes with a large-scale deployment. Some participants attended this workshop just to learn which sensors would be most widely recommended and free of errors or issues. Several received the idea of a data repository with enthusiasm, partly because the burden of creating and hosting a database could be lifted.

### 4.4. Data Interpretation Needs

Researchers working with low-cost air quality sensors are generally aware of the quality and uncertainty associated with their sensor measurements. Many other users, especially more casual users, may need more assistance in understanding the limitations of the technology and interpretation of the data. Repeatedly, practitioners mentioned the need for effective infographics to help share data, interpret the results, and educate the public.

Experts in epidemiological research, including participants Rima Habre (University of Southern California, Los Angeles, CA, USA) and Michael Jerrett (University of California, Los Angeles, CA, USA), noted that low-cost air quality sensors are changing the type of exposure data available often pushing toward a goal of measuring personal exposures. Highly time-resolved data (seconds to minutes) creates opportunities for new research in deciphering the impact of acute exposures to various pollutants. Numerous researchers are exploring the development of apps and websites aimed at helping people explore and interpret their personal exposures. Given the current state of sensor science, with relatively large measurement uncertainties, there is a concern from practitioners about encouraging citizens to change behavior based on sensor measurements. On the one hand, users observing spikes may be prompted to change their behavior resulting in reduced exposure. On the other hand, the reduced exposure may not result in an observable health outcome and users may be less likely to continue with their behavior changes as a result. Moreover, spikes in air quality data may result in users experiencing increased stress, possibly negating any other health benefit.

### 4.5. Sensors and Modeling

One of the motivations for measurements with low-cost sensors is to increase the spatial resolution of our atmospheric measurements to identify variation below the city or regional level, even down to the city block-level or below [40]. Current modeling techniques struggle at this level due to the high dynamic variability of pollution sources, wind, obstructions, etc. [41]. Because of the greater uncertainty associated with the low-cost sensor measurements, much work is still needed to determine if low-cost sensing can improve model performance and better describe personal exposure. There are some on-going efforts using machine learning to inform models with low-cost sensor measurements [42,43].

## 5. Panel 4 and a Community Panel: Lessons Learned from Research/Community Partnerships

Communities are demanding a greater role in scientific research and decision-making that impacts their lives. Across the US and globally, residents continue to recognize that pollution sources impact their neighborhoods and exposure to pollutants may be causing health hazards for them based on where they live, work, and play. Further, communities are increasingly seeking tools to document these exposures and environmental health disparities. Currently, regulatory air monitoring systems generally do not assess neighborhood variability in air quality at a sufficiently refined spatial scale [44,45]. The increase in the availability of low-cost air pollution sensors has increased the number of citizen scientists collecting and using air quality data to better characterize and understand their local environment. Education and involvement of communities in science and research is not only important for improving public health; it is also important for building awareness about the sources of air pollution, exposure pathways, and the association between contaminants and health endpoints [46]. During Panel 4, panelist Jill Johnston (University of Southern California, Los Angeles, CA, USA), Nicole Wong (Redeemer Community Partnership, Los Angeles, CA, USA), Vanessa Galaviz (State of California, OEHHA, Oakland, CA, USA), Ashley Collier (University of Colorado, Boulder, CO, USA), and Andrea Clements (U.S. Environmental Protection Agency, Research Triangle Park, NC, USA) outlined key aspects to forging a successful partnership with communities that are seeking information about ambient air quality and offered recommendations for how low-cost sensors can be deployed for research by citizen scientists. In addition, community leaders Sandy Navarro (People not Pozos, Los Angeles, CA, USA) and Luis Olmedo (Comite Civico Del Valle, Inc., Brawley, CA, USA) shared specific examples from their experiences as community leaders addressing air quality concerns (see Section 5.3 for more details about these community projects).

### 5.1. Building Scientific Literacy

Low-cost air quality sensors offer new opportunities to gather data about local air quality in an individual home, during a bike ride, or in various neighborhood parks simultaneously. Devices that measure real-time pollution and provide immediate feedback have the opportunity to serve as tools to build the capacity of residents to understand air pollution, spatial and temporal variability, and exposure patterns relevant to their community. Through this process, residents can learn about scientific methods, the ability to interpret data within a given context, and the potential links between air quality and health outcomes [47]. Residents offer expertise to identify potential sources of air pollutants otherwise unknown to scientists or regulators as a result of their lived experiences and knowledge of their neighborhood. Collaboration and bidirectional dialogue is important to characterize the question, evaluate whether available low-cost sensors are appropriate for addressing that question, and design a method for collecting the data. Community members also contribute observational data or qualitative information to add context to recorded pollutant concentrations. It is key, however, that all parties understand both the advantages and limitations of low-cost sensing.

### 5.2. Leveraging Low-Cost Sensors

Low-cost sensor technology can be leveraged to advance the co-production of knowledge. During the initial phases of study design, a variety of expertise should be considered, such as that of community members, scientists, regulatory partners, and even representatives from potential sources of concern. Academic or regulatory partners can support communities to ensure appropriate sensor technologies are chosen considering: (1) the pollutant or source of interest, (2) spatial and temporal scale of interest, and (3) the “ease” of interpretability of the data. Collaborators should discuss the design of defensible calibration techniques and collocation with regulatory monitors. Community members offer vital knowledge about important factors that may need to be considered when planning how to conduct observations of the problem (source, frequency, intensity, etc.) [48], and possess vital community contacts to help with community engagement.

In order to build trust between community members, scientists, and regulatory bodies, panelists and attendees made several best-practice recommendations:

- Discuss, during the study design phase, responsibilities and expected outcomes with all key partners.
- Explain the capabilities of sensor measurements at the time of a partnership and educate all parties on the current challenges that remain for the field.
- Clarify the expectations of what sensor data can and cannot help elucidate, how the sensor data compares to “gold standard” FRM/FEM instruments, and what will happen with the data during and after the study. Such agreements should all be clearly outlined and accepted by partners.
- Outline the limitations of such data for use by regulatory agencies.
- Prepare residents for various potential outcomes based on their questions, such as negative or no results.
- Establish agreements regarding data sharing and ownership, communication of results, and publication during the study design phase.

Collaborative teams may find it valuable to include social scientists or to look to other disciplines for examples of useful formats for sharing data/results [49], effective ways of communicating risk [50], or the principles of community-based participatory research (CBPR) established in public health research [51].

### 5.3. Opportunities for Community-Driven Science

Community-research partnerships can prompt action to prevent harmful exposures or improve local air quality. Innovators continue to advance low-cost sensor technology, but even with the existing limitations, sensor systems on the market now can still provide insight for communities aiming to gather data about ambient air exposures. For example, while exact concentration measurements may be fairly uncertain, relative difference within or between communities or before and after an event (e.g., engine changeover or reactivation of industrial source) may still be valuable depending on the questions and goals of a particular community. Similarly, sensors may be able to give insight into spatial/temporal patterns as well as determine “hotspots” for future targeted studies with more sophisticated instrumentation.

During the workshop, two community leaders shared their thoughts on applications for and experiences with low-cost sensors. Sandy Navarro from People Not Pozos, a grassroots program that is part of Esperanza Community Housing based in South Los Angeles, described using sensors as tools to better identify local air exposures. Since 2010, local residents have complained of noxious odors and health symptoms (e.g., respiratory illness, fatigue, headaches, nausea, eye & throat irritation, dizziness, and spontaneous nosebleeds) [52]. Many of those residents identified a nearby oil drilling site, situated across the street from one of Esperanza’s low-income housing buildings, as a source of odors and air pollution. After three years of official complaints and protests by this environmental justice community, investigators from the USEPA visited the site and discovered violations resulting in a shutdown of operations [53]. People Not Pozos organized in response to this issue; including collaboration with researchers in order to collect environment and health data and training community residents to engage as researchers on the project. Nonetheless, community frustrations persist. The community has not received response to official complaints, sufficient data or access to collected data, nor easily understandable information in an accessible way (e.g., Spanish translations). Thus, an on-going project, in collaboration with another group of researchers, has deployed a small network of low-cost sensors to characterize neighborhood-scale air quality.

The subsequent discussion highlighted a common problem in public health and environmental justice: that need is often greater than the capacity. Particularly in a city like LA, given its size and density, public health officials are likely to be limited by available resources (including both equipment and time). A researcher from the local regulatory agency expressed precisely this sentiment, indicating that the agency is piloting new technologies to try and help expand their capacity to investigate community concerns and complaints. Among these new technologies are low-cost sensor systems, which may be able to serve as a sort of alarm. Additionally, he noted that communication is an issue and it’s likely the residents’ complaints and requests for data were probably not reaching the appropriate person. This example highlights an area where local regulatory agencies can assess the effectiveness of or improve their education and outreach efforts to the communities they serve.

In the Imperial Valley, Luis Olmedo with the nonprofit Comite Civico Del Valle shared another story describing the Identifying Violations Affecting Neighborhoods (IVAN) monitoring system. This system includes a platform for submitting and viewing environmental reports, as well as real-time data from a network of 40 air quality monitors utilizing low-cost particulate matter sensors [54]. On the website, users can receive air quality alerts that include recommendations for adjusting outdoor physical activity to reduce an individual’s exposure. These recommendations are based on a scale that the IVAN team has developed that provides numeric and color indicators based on PM concentrations and potential health impacts [55]. In the Imperial Valley, this system has been integrated into a school-based flag program that uses colored flags to indicate air quality and provides recommendations on outdoor activity. While recognizing this information is still limited, Luis asserted that this strategy enables individuals to make their own choices about their health and potential exposures. These monitors provide a picture of PM concentrations at a spatial scale and resolution previously unavailable allowing the community to take targeted action. Actions that are especially important given that Imperial County has the highest rates of asthma-related hospitalizations and emergency room visits among school-aged children of all counties in California [56].

There remains a need to develop best practices for risk communication and visualization of air sensor data for residents. For example, in the case of real-time data, it is important to communicate the difference between short-term high exposures versus 24-h or weekly averages in pollutant levels. The use of real-time and personal monitoring with low-cost sensor provides an opportunity to better assess dose-response relationships to various health outcomes and more specifically study vulnerable and susceptible populations—such as asthmatics or those living in environmental justice neighborhoods. Ultimately, the results from sensor studies have the potential to help communities decide on actions they themselves wish to take to protect health [57].

### 5.4. Summary

Overall, community-driven research using sensors is likely to benefit both community and scientists alike. In particular, the communities facing the greatest environmental exposure risks and health effects are demanding a greater role in researching, describing and prescribing solutions to address the local environmental hazards they face [58,59]. Coupled with technical expertise and air quality sensors, communities can play a central role in defining the problems, supplying local knowledge and interpreting the results in the context of the local reality. With communities’ expertise, there are improvements in the relevance of research questions at the scientific level. Community research may also help to build trust and empower participants and community members, especially when the data is community owned and managed, giving them a ‘seat at the table’ with industry and regulators. Moreover, using sensors in community science allows for real-world application of the research, allowing for people to make a difference and improve the health and lives of their community members.

## 6. Stakeholder Small Group Discussions

To conclude the workshop, attendees split into small groups to discuss what could be taken away from the workshop discussions. Attendees were given general guidance to focus on three core topics: existing resources to be shared and new resources that should be developed, important takeaways or best-practices that could be shared more widely, and important next steps for the field. A theme that emerged from these discussions was the need to improve communication between all stakeholders and how communication strategies could address the challenges highlighted in this paper. The variety of stakeholders and accelerating pace of research necessitate a variety of communication strategies to address the breadth of challenges in this field. In general, the group discussions focused on communications between and within two main groups of stakeholders: researchers and the participating community members.

### 6.1. Creating Dialogue in Community-Based Research 

Communication between researchers using low-cost air quality sensors and engaged members of communities in which those sensors are deployed is both challenging and vitally important. A key challenge is creating a dialogue that brings all stakeholders to the table and values each member’s knowledge and perspectives. Successfully establishing this dialogue will improve project relevance and data quality while identifying other areas of interest that might otherwise be overlooked.

Establishing realistic expectations between all parties at the onset of community-based research studies is paramount. Sensor limitations must be discussed and the measurements adequately contextualized. The community’s concerns, objectives, and insights must be discussed. Some collaborations have found it helpful to establish Memoranda of Understanding (MoU) and/or Frequently Asked Question (FAQ) pages for their projects. All parties involved in the research should collaboratively develop such documents so that they are easy to understand (including language translation when necessary) and adequately capture the expectations and responsibilities of all project partners. Developing standard templates for and promoting the wider use of MoU in community-based research projects could help maintain positive relationships and create engaged communities that are more open to working with researchers.

An important expectation that should not be overlooked is the ownership and control of data collected during a community-based research project. Every community’s expectations will be different and researchers should be mindful of the sense of ownership that community members may feel toward data that they were responsible for collecting. Researchers should share data in a manner that respects the wishes of the community in which it was collected.

Appropriately communicating data can be especially challenging given the developmental nature of these instruments. However, it is critical to develop data communication methods that allow community members to transparently access and understand data and uncertainty while providing adequate context. Enhancing access to the data will allow researchers and community partners to collaboratively draw insights from the data. These methods may come in a variety of forms and the communication of data to non-technical persons should also be a consideration when designing infographics and interpretation or visualization tools for community based research. By developing best practices for data visualization and communication, researchers can help communities to better quantify and communicate aspects of their lived reality.

### 6.2. Collaboration and Standards for Low-Cost Sensor Research 

As research in the field of low-cost sensors accelerates, it will become increasingly important for researchers to harmonize terminology and data reporting formats. One effort that was widely discussed at the close of the workshop was the Data Platform initiative being led by the EDF (discussed in Section 3). This project attempts to create a database and schema that would allow users to openly share data collected using low-cost air quality sensors. A lengthy discussion focused on how standardizing a data reporting format based on sensor type could facilitate largescale comparison between research projects and allow researchers to test calibration models on a larger parameter space. The formatting guidelines could include details as simple as the date and time string format to the specific metadata that should be included.

Given interest in large scale comparisons between research projects, it is important that metadata include information about the sensor and its performance and measurement uncertainty. Most importantly, data quality elements such as precision, bias, detection limit, age, and calibration can be imbedded in the metadata. This information can give researchers more information about the measurement uncertainty and help them determine the usefulness of the data for a given application. It could also streamline collaborations between projects using a variety of different instruments and more generally improve communications between stakeholders.

Calibration presents another complicated challenge for communication and comparison. For example, when researchers compare different calibration techniques, it is important to be mindful of the statistical techniques used in these comparisons (e.g., RMSE, R-squared, correlation coefficient). It is likely that the best metric to compare and select a calibration model will depend on the application and goals of the project. The difficulty in fairly evaluating calibration methodologies, including physical methodologies and numerical methods was discussed at length.

Physical methodologies, like collocation, have questionable applicability once sensors are moved into a new area to collect data. Once collocation calibration data has been collected, a variety of numerical calibration methods are currently utilized ranging from simple linear fits to complex machine learning and artificial neural networks [8,11,12,31,32,60].

From the workshop discussions, it was clear that additional research is needed to develop a comprehensive best practice for sensor calibrations. One idea for calibration method comparison could include sharing a collection of data from a variety of sensor technologies in a number of unique environments that include collocated reference data. These datasets could allow researchers to test novel methodologies and to compare their effectiveness against previous methods. This data could be released as sets of “training” datasets including reference data and “validation” sets without reference data to penalize over-tuning of calibration algorithms.

### 6.3. Going Forward

The low-cost air quality sensing workshop provided an excellent forum for researchers, regulators, manufacturers, and community advocates to discuss a sampling of the challenges and successes in this fast-growing field. Going forward, it will be important to continue to hold workshops like this and to include an even broader group of stakeholders in the discussions. This may include policy makers, electrical engineers, programmers, and others from both the United States and abroad. With such a fast-moving state of technology, it will remain important to collaborate with all parties to ensure that research projects are successful and relevant. Beyond workshops, there was also interest expressed in other methods of staying connected and sharing resources, for example via a listserv or wiki page. Toward that goal, Table 1 shares a list of existing resources that were compiled during the final discussion. This list is not comprehensive but may serve as a starting point. These are valuable resources for those involved in air quality and environmental justice and sharing others not listed here is equally important. Please note that inclusion or omission does not indicate an endorsement or lack thereof of these tools.

## 7. Workshop Conclusions

This workshop provided the opportunity to reflect on the current state of low-cost air quality sensor research. The discussions made it clear that better communication within the field could help integrate the wide array of knowledge held by researchers, communities, and other stakeholders. There was consensus among the diverse group of attendees that, for the time-being, low-cost air-quality sensing was a complement, not a replacement for high-end sensing. Likewise, attendees agreed that to properly utilize low-cost sensing, it is critical to apply sensors in ways that complement the capabilities of the sensors. The group also expressed optimism that despite sensor limitations there are areas in which studies using low-cost sensors can make valuable contributions. In the next phase of low-cost air quality sensor research, a goal should be the bringing together of diverse sets of expertise to identify and tackle ongoing and emerging issues, especially via projects that lead to data driven actions and improved public health.

## Figures and Tables

**Table 1:** Online tools that may be useful to low-cost air quality research stakeholders.

Category	Tool	Description	Website
Government	EPA’s Air Sensor Toolbox	Resources to support communities and citizens selecting and using low-cost sensors	epa.gov/air-sensor-toolbox
	AirNow	Aggregated national data from regulatory grade monitoring stations and information on potential health impacts	airnow.gov
	EJSCREEN	Environmental justice screening and mapping tool	epa.gov/ejscreen
	RETIGO	EPA software for mapping collected air quality data	epa.gov/retigo
	C-FERST	Community-focused exposure and risk screening tool	epa.gov/c-ferst
	AQ-SPEC	Sensor evaluation program	aqmd.gov/aq-spec
	US EIA Energy Mapping System	Mapping tool to explore energy use and development across the United States	eia.gov/state/maps.php
	CalEnviroscreen	California specific environmental justice screening tool	oehha.ca.gov/calenviroscreen
Non-Profit and Academic	IVAN Monitoring System	Community-based monitoring system for environmental concerns	ivan-imperial.org/air
	FrackTracker	App for logging reports and observations around oil and gas activity	fractracker.org
	Institute for Health and Metrics Evaluation	Resource for exploring health data and statistics	healthdata.org
	EDF Air Sensor Workgroup Data Standards	Guidelines for sensor data formatting	edf.org/health/data-standards-date-and-timestamp-guidelines
	AQ-IQ Air Quality and Sensor Curriculum	K-12 curriculum supporting the use of low-cost sensors by students	teachengineering.org/curricularunits/view/cub_airquality_unit
	OpenAQ	Aggregated global data from regulatory grade monitoring stations	openaq.org
	SensorThingsAPI	Open source API for connecting IoT sensing devices, data, and applications over the web	opengeospatial.org/standards/sensorthings
Commercial	Envirosuite	Software for environmental management and planning	envirosuite.com
	Breezometer	Commercial API for displaying air quality indicators	breezometer.com
